# Galectin-4 is associated with diabetes and obesity in a heart failure population

**DOI:** 10.1038/s41598-023-47426-9

**Published:** 2023-11-20

**Authors:** Anna Dieden, Petri Gudmundsson, Johan Korduner, John Molvin, Amir Zaghi, Zainu Nezami, Erasmus Bachus, Hannes Holm, Amra Jujic, Martin Magnusson

**Affiliations:** 1 Department of Clinical Sciences Malmö, Lund University, Malmö, Sweden; 2 Department of Biomedical Science, Malmö University, Malmö, Sweden; 3 Biofilms- Reseach Centre for Biointerfaces, Malmö University, Malmö, Sweden; 4 Late-Stage Development, Cardiovascular, Renal, and Metabolism, BioPharmaceuticals R&D, AstraZeneca, Gothenburg, Sweden; 5 Department of Cardiology, Skåne University Hospital, Malmö, Sweden; 6 Hypertension in Africa Research Team (HART), North West University, Potchefstroom, South Africa; 7 Wallenberg Center for Molecular Medicine, Lund University, Lund, Sweden

**Keywords:** Biomarkers, Cardiology, Endocrinology

## Abstract

An association between high Galectin-4 (Gal-4) and prevalence of diabetes in subjects with heart failure (HF) has previously been reported. The purpose of this study was to confirm these findings, as well as to further investigate this association, in a Swedish HF population. In addition, a second aim was to explore Gal-4’s association with obesity and biomarkers of metabolism and heart failure. Gal-4 was measured using a proximity extension array technique in 324 hospitalized HF patients within the Swedish HeArt and bRain failure investigation trial cohort. Obesity was defined as BMI ≥ 30. Multivariable logistic regression models were used to explore associations between Gal-4 and diabetes/obesity, and linear regression models were used to explore the associations between Gal-4 and biomarkers. A total of 309 participants (29.1% female; mean age 74.8 years) provided complete data for the analysis of associations between Gal-4 and diabetes. Additionally, for the analysis of heart failure phenotype, complete data was available for 230 subjects. Gal-4 was positively associated with prevalent diabetes (OR 2.60; CI 95% 1.56–4.32). In multivariable models, Gal-4 levels were significantly associated with obesity, but only for subjects with diabetes (OR 2.48; 1.09–5.62). Additionally, Gal-4 demonstrated a significant association with the incretin Glucose-dependent insulinotropic polypeptide (GIP), as well as with biomarkers of HF. In the stratified analyses, the association between Gal-4 and diabetes was prominent in patients with reduced ejection fraction (n = 160, OR 3.26; 95%CI 1.88–5.66), while it was not observed in those without (n = 70, 1.96 (0.75–5.10)). In this cross-sectional, observational study, higher Gal-4 levels in HF patients were associated with higher GIP levels. Further, increased levels of Gal-4 were associated with increased likelihood of diabetes, and obesity. This association was particularly pronounced in individuals with HF characterized by reduced ejection fraction. Additionally, Gal-4 levels were significantly elevated in heart failure patients with diabetes and obesity.

## Introduction

The prevalence and incidence of heart failure (HF) in people with diabetes is very high and mortality as well as risk of re-hospitalization due to HF is much greater in individuals with diabetes, compared to those without^1^. Conversely, the risk of diabetes is increased in individuals with HF^2^. In a recent study of a multinational cohort of 9,428 HF outpatients the prevalence of diabetes was almost as high as 40%^3^. However, the treatment of diabetes in patients with concomitant HF is complex as use of several commonly used diabetic medications have shown to affect the risk of adverse events in observational studies and randomized controlled trials^4,5^. To improve risk stratification and optimize treatment within the area, there is an urgent need of deeper understanding of the pathophysiological pathways related to diabetes and HF.

Galectin-4 (Gal-4) is a part of the galectin family of 15 small lectin proteins and is expressed almost exclusively in the gastrointestinal tract of healthy subjects. This has made it an interesting candidate as a cancer marker since it is induced by several malignancies^6^. Gal-4 performs several functions, including cell adhesion and induction of intracellular signaling^6,7^. Another function of Gal-4 is the stabilization of lipid rafts for the apical transport of proteins from the Golgi apparatus to the apical membrane of the enterocyte^8^. This is interesting because one of the transported proteins is the protease dipeptidyl peptidase-4 (DPP4) which is well known for cleavage and inactivation of our two most common incretins: glucose-dependent insulinotropic polypeptide (GIP) and proglucagon-derived peptide glucagon-like peptide-1 (GLP-1). The inactivation of GIP and GLP-1 by DPP-4 leads to several cardiometabolically adverse effects, including endothelial dysfunction, insulin resistance, and hyperlipidemia^9^. There is an established association between diabetes and pathways related to inflammatory response, extracellular matrix components and cardiac fibrosis, and studies have revealed Gal-4’s association with HF^10^ and diabetes^11–15^. Studying obese subjects in a general population, we have previously reported an association between higher levels of Gal-4 and obese subjects with a history of hospitalization and that this association was only significant in subjects with diabetes^12^. Further, we have also previously shown an association between higher levels of Gal-4 and prevalent, as well as incident, diabetes in a single population cohort study^11^. In women with gestational diabetes mellitus (GDM), levels of Gal-4 were increased in the placenta, compared to subjects without GDM^13^. As for Gal-4’s association with HF, Bouwens et al. could reveal a strong association between higher levels of Gal-4’s, as well as its increased change over time, and adverse outcome in HF patients^16^. Another study revealed similar results as Gal-4 levels were higher in patients with severe HF, as compared to controls, and were also associated with all-cause mortality^17^. In addition, in a general population, we have previously shown that high levels of Gal-4 was associated with incident HF, as well as cardiovascular disease (CVD) and mortality^10^.

However, studies investigating Gal-4’s role in concomitant diabetes and HF are scarce and to the best of our knowledge, no studies have explored the association between Gal-4 and obesity. Using multiplex proteomics, an association between high Gal-4 and the prevalence of diabetes in subjects with HF has previously been reported^18^. The purpose of this study was to confirm these findings, and to further investigate this association as well as Gal-4’s association with obesity and incretin hormones, within a Swedish HF population. Moreover, considering the heightened myocardial fibrosis observed in individuals with diabetes, a condition attributed to various biological and molecular mechanisms^19,20^, we conducted stratified analyses to investigate the associations between Gal-4 and HF aetiology and phenotype.

## Methods

### Study population

The HeArt and bRain failure inVESTigation trial (HARVEST-Malmö)^21^ is an ongoing, prospective cohort study in Malmö Sweden that started in 2014. The only inclusion criterion is an admission to a cardiological or internal medicine ward for treatment of HF at the public tertiary care hospital. The only exclusion criterion is an inability to give informed consent. If patients have severe cognitive impairment, their relatives are informed about the study, and asked for the permission on the patient’s behalf. Between March 2014 and January 2018, 324 consecutive patients hospitalized for newly onset or worsening HF were enrolled in the study and Gal-4 levels were analysed using a proximity extension array technique. One patient was excluded due to Gal-4 levels being an outlier and of the 323 patients remaining, 309 had complete data on all covariates for analyses of Gal-4’s associations with diabetes and obesity. For analyses of associations between Gal-4 and HF aetiology and HF phenotype, data was available for 305 and 230 subjects, respectively.

### Various definitions/clinical examination

Diabetes was defined by the presence of one or more of the following criteria: self-reported physician diagnosis of type 2 diabetes, use of diabetic medication, or fasting plasma glucose (FPG) levels equal to or greater than 7 mmol/L. Systolic blood pressure measurements were obtained following a 10-min rest period in the supine position. Body mass index (BMI) was calculated as kg/m^2^, and obesity was defined as having BMI ≥ 30. Self-reported physical activity was acquired using a questionnaire, and a sedentary lifestyle was defined as ≤ 1 h exercise in a normal week. NYHA-class was assessed by the attending physician. Ischemic heart disease (IHD) was defined as a previous myocardial infarction, or myocardial infarction at study inclusion. HF with preserved ejection fraction (HFpEF) was defined as a left ventricular ejection fraction (EF) ≥ 50%, while HF with reduced ejection fraction (HFrEF) was defined as EF < 50%. Left ventricular hypertrophy (LVH) was defined as left ventricular mass/body surface area (LVMI) ≥ 115 g/m2 in men, or ≥ 95 g/m2 in women.

### Echocardiography

All examinations were carried out by experienced sonographers using a Philips IE333 with a 1–5 MHz transducer, or a GE Vingmed Vivid 7 Ultrasound with a 1–4 MHz transducer for transthoracic echocardiograms. Standard views (parasternal long axis, apical four-chamber, and two-chamber) were used to obtained cine loops. LVEF was calculated automatically from end-diastolic volumes (EDV) and end-systolic volume (ESV) (EF = (EDV − ESV)/EDV). The parasternal long-axis view was used to assess the internal dimensions of both the left and right ventricles at end-diastole. Additionally, measurements of wall thickness were taken from a two-dimensional end-diastolic parasternal long-axis view. Left ventricular mass was calculated using Devereux's formula.

### Laboratory analyses

Fasting blood samples were collected in the morning following study inclusion. Cystatin C, fasting plasma glucose (FPG), triglycerides, serum insulin and N-terminal pro–B-type natriuretic peptide (NT-proBNP) were analysed at the Department of Clinical Chemistry, Malmö. The plasma level of cystatin C was determined by an automated particle-based immunoassay, using the Hitachi Modular P analysis system and reagents from DAKO (Dako A/S, Glostrup, Denmark). Plasma triglycerides and FPG were analysed using Cobas c501/Cobas c701 (Roche, Basel, Switzerland). Total serum GIP was analyzed using Millipore’s Human GIP Total ELISA (cat. No. EZHGIP-54 K). Serum concentrations of insulin were analyzed using Cobas 6000/8000 (Roche, Basel, Switzerland). Aliquots (225 µL; REMP, Brooks, Life Sciences, USA) of plasma were stored in − 80 °C in a local biobank in the Region Skåne County Council until proximity extension array analyses (May 2018). Plasma concentrations of Gal-4, Galectin-3 (Gal-3), and Suppression of tumorigenicity 2 (ST2) were determined through a proximity extension array technique, using Proseek Multiplex CVD III 96 × 96 reagents kit (Olink, Uppsala, Sweden). The final proteomic assay read out was given as an arbitrary unit given on a log2 scale meaning that each unit increase corresponds to a doubling in concentration. Homeostatic Model Assessment for Insulin Resistance (HOMA-IR) was calculated using the formula: HOMA-IR = (fasting insulin (µU/ml) x fasting glucose (mmol/L)/22.5.

### Statistical analysis

The variables are reported as means (± standard deviation (SD)) or median (25–75 interquartile range). Continuous variables were compared using one-way analysis of variance (ANOVA) tests for normally distributed variables, or Mann Whitney U-test for non-normally distributed variables, while binary variables were assessed using χ2 tests. Prior to analysis, variables with a non-normal distribution (Cystatin C, FPG,triglycerides and HOMA-IR) were ln-transformed. Initially, we conducted univariate linear regression analyses to investigate the associations between Gal-4 and FPG, HOMA-IR, GIP, ST2, NT-proBNP, and Gal-3, respectively. Subsequently, significant associations were further adjusted for age and sex. To explore the association between Gal-4 levels and diabetes, logistic regression was used, initially unadjusted, followed by adjustment according to Model 1 (age and sex), and further adjusted according to Model 2a (triglycerides, FPG, BMI, systolic blood pressure, cystatin C and physical activity). Further, we explored the associations between the quartile with the highest levels of Gal-4 (upper quartile), compared to all other quartiles, and diabetes, using the same approach as described above, where upper Gal-4 quartile replaced Gal-4. In the next step, we explored Gal-4’s unadjusted associations with obesity, followed by adjustment for Model 1, and further adjustment according to Model 2b (triglycerides, FPG, systolic blood pressure, cystatin C and physical activity). The same approach was used to analyse the upper quartile of Gal-4 compared to all other quartile’s association with obesity. Following an interaction analysis with diabetes as a mediator variable in analyses of Gal-4’s association with obesity, a post-hoc stratified analysis was carried out, dividing the population into individuals with or without diabetes and thereafter exploring Gal-4’s association with obesity.

In the next step, Gal-4’s associations with (a) HF aetiology (ischemic/non-ischemic HF), (b) HF phenotype (HFrEF/HFpEF), (c) functional capacity (NYHA-class) and structural cardiac changes (LVH), were explored in unadjusted logistic regression, where significant findings were taken further to Model 1 and Model 2a.

Gal-4's association with diabetes and obesity was thereafter explored unadjusted, in Model 1, and in Model 2a (diabetes) or Model 2b (obesity) separately in groups stratified on a) IHD/non-IHD, and b) HFpEF/HFrEF.

Analyses were performed using IBM SPSS Statistics v. 28 (Chicago, Illinois) and statistical significance was determined based on a two-sided *p*-value of less than 0.05.

### Ethics approval and consent to participate

The study was approved by the Ethical Review Board at Lund University, Sweden and it fulfills the Declaration of Helsinki. A written informed consent was obtained from all participants or relatives.

## Results

### Study population characteristics

The study population is described in Table 1. Individuals with diabetes had significantly higher levels of Gal-4, higher prevalence of IHD, higher BMI, higher levels of GIP, triglycerides, cystatin c, and FPG, along with higher HOMA-IR, compared to individuals without diabetes. Between the two groups, no differences were seen in age, sex, systolic blood pressure, physical activity and LVH.

**Table 1:** Baseline characteristics.

	All subjects (n = 309)	Individuals with diabetes (n = 115)	Individuals without diabetes (n = 194)	p	Individuals with obesity n = 97	Individuals without obesity n = 212	p
Clinical							
Age (years)	74.8 (± 11.5)	74.4 (± 9.0)	75.0 (± 12.8)	0.662	70.4 (± 10.4)	76.8 (± 11.5)	** < 0.001**
Sex (female, %)	90 (29.1)	28 (24.3)	62 (32.0)	0.155	32 (33.0)	58 (27.4)	0.312
BMI (kg/m^2^)	27.9 (± 5.8)	30.5 (± 5.7)	26.4 (± 5.3)	** < 0.001**	34.8 (± 4.5)	24.8 (± 2.9)	** < 0.001**
SBP (mmHg)	136.8 (± 27.8)	136.1 (± 26.2)	137.2 (± 28.8)	0.737	142.6 (± 30.1)	134.1 (± 26.4)	**0.013**
Physical activity (sedentary, %)	182 (58.9)	69 (60.0)	113(58.2)	0.762	56 (57.7)	126 (59.4)	0.778
Diabetes (n (%))	115 (37.2)	–	–	–	60 (61.9)	55 (25.9)	** < 0.001**
IHD (n (%))	126 (41.3) [n = 305]	62 (55.0) [n = 112]	64 (33.1) [n = 193]	** < 0.001**	44 (45.4) [n = 97]	82 (39.4) [n = 208]	0.327
LVH (n (%))	150 (65.5) [n = 229]	57 (67.9) [n = 84]	93 (64.1) [n = 145]	0.568	52 (69.3) [n = 75]	98 (63.6) [n = 154]	0.395
Medical							
ARB	80 (25.9)	32 (27.8)	48 (24.7)	0.550	26 (26.8)	54 (25.5)	0.804
ACE-i	164 (53.1)	60 (52.2)	104 (53.6)	0.807	50 (51.5)	114 (53.8)	0.716
Betablocker	271 (87.7)	103 (89–6)	168 (86.6)	0.443	89 (91.8)	182 (85.8)	0.143
Laboratory							
Triglycerides (mmol/L)	1.0 (0.8–1.3)	1.1 (0.8–1.4)	1.0 (0.8–1.2)	**0.007**	1.1 (0.9–1.5)	1.0(0.8–1.3)	**0.003**
Cystatin C (mg/L)	1.7 (1.3–2.1)	1.8 (1.5–2.3)	1.6 (1.3–2.1)	**0.005**	1.6 (1.3–2.0)	1.7 (1.3–2.2)	0.412
Fasting plasma glucose (mmol/L)	6.2 (5.4–7.5)	7.6 (6.6–9.6)	5.8 (3.8–6.5)	** < 0.001**	7.2 (6.0–8.8)	6.0 (5.4–7.0)	** < 0.001**
GIP (pg/ml)	76.1 (52.3–113.5) [n = 284]	87.2 (59.6–124.4) [n = 176]	70.6 (49.3–100.5) [n = 108]	**0.003**	85.2 (61.9–128.4) [n = 92]	69.8 (49.3–104.5) [n = 192]	**0.003**
NT-proBNP (pg/mL)	4112.0 (2246.0–8774.8) [n = 308]	3820.0 (2116.0–7892.0) [n = 115]	4141.0 (2299.0–8826.0) [n = 193]	0.455	2861.0 (1281–5582.5) [n = 97]	4870.0 (2701.0–10,699.0) [n = 211]	** < 0.001**
Insulin (µU/ml)	7.0 (4.0–11.0) [n = 296]	8.0 (3–12) [n = 111]	7.0 (4.0–11.0) [n = 185]	0.634	10.0 (8.0–16.0) [n = 95]	6.0 (4.0–9.0) [n = 201]	** < 0.001**
HOMA-IR	2.1 (1.1–3.3) [n = 296]	2.6 (1.2–4.9) [n = 111]	1.9 (1.1–3.1) [n = 185]	**0.006**	3.4 (2.2–6.1) [n = 95]	1.7 (1.0–2.6) [n = 201]	** < 0.001**
Gal-4 (AU)	4.0 (± 0.6)	4.3 (± 0.6)	3.9 (± 0.6)	** < 0.001**	4.1 (± 0.7)	4.0 (± 0.6)	**0.021**

Significance values are in bold.

Values are means and (± standard deviations), medians and (25–75 interquartile range), or numbers (percentages).

*BMI* body mass index, *SBP* systolic blood pressure, *IHD* ischemic heart disease, *LVH* left ventricular hypertrophy, *ARB* Angiotensin receptor blockers, *ACE-i* angiotensin-converting-enzyme inhibitors, *GIP* Glucose-dependent insulinotropic polypeptide, *NT-proBNP* N-terminal pro–B-type natriuretic peptide, *HOMA-IR* homeostatic model assessment for insulin resistance, *Gal-4* Galectin-4, *AU* arbitrary units.

Individuals with obesity had higher levels of Gal-4, were older, had a higher BMI, higher levels of NT-proBNP, GIP, triglycerides, insulin, and FPG, along with higher HOMA-IR, compared to individuals without obesity. Between the two groups, no differences were seen in sex, cystatin c, physical activity and LVH. Moreover, upon stratification of the cohort into two distinct groups by IHD aetiology (n = 305), it was observed that individuals with a history of IHD exhibited higher levels of Gal-4 in comparison to those without (4.12 (± 0.59) vs 3.96 (± 0.66), *p* = 0.024).

In linear regression analyses, Gal-4 was significantly positively associated with FPG, GIP, ST2, NT-proBNP and Gal-3 in a model adjusted for age and sex, but not with HOMA-IR (Table 2).

**Table 2:** Associations between Gal-4 and biomarkers of metabolism and heart failure.

	FPG		HOMA-IR		GIP		ST2		NT-proBNP		Gal-3	
Unadjusted	B	P	B	P	B	p	B	P	B	P	B	P
Gal-4	0.062	**0.012**	0.117	0.195	0.213	** < 0.001**	0.435	** < 0.001**	0.756	** < 0.001**	0.415	** < 0.001**
Model 1												
Gal-4	0.060	**0.017**	0.155	0.091	0.233	**0.001**	0.426	** < 0.001**	0.646	** < 0.001**	0.394	** < 0.001**
Age	0.001	0.716	− 0.009	0.080	− 0.005	0.120	0.003	0.553	0.028	**0.001**	0.006	**0.010**
Sex	0.006	0.864	0.316	**0.016**	0.131	0.084	0.085	0.439	− 0.537	**0.011**	0.128	**0.022**

Significance values are in bold.

Values are unstandardized beta coefficients.

*FPG* fasting plasma glucose, *HOMA-IR* homeostatic model assessment for insulin resistance, *GIP* Glucose-dependent insulinotropic polypeptide, *NT-proBNP* N-terminal pro–B-type natriuretic peptide, *ST2* Suppression of tumorigenicity 2, *Gal-3* Galectin-3, *Gal-4* Galectin-4.

### Gal-4’s association with diabetes and obesity

**Figure 1:**
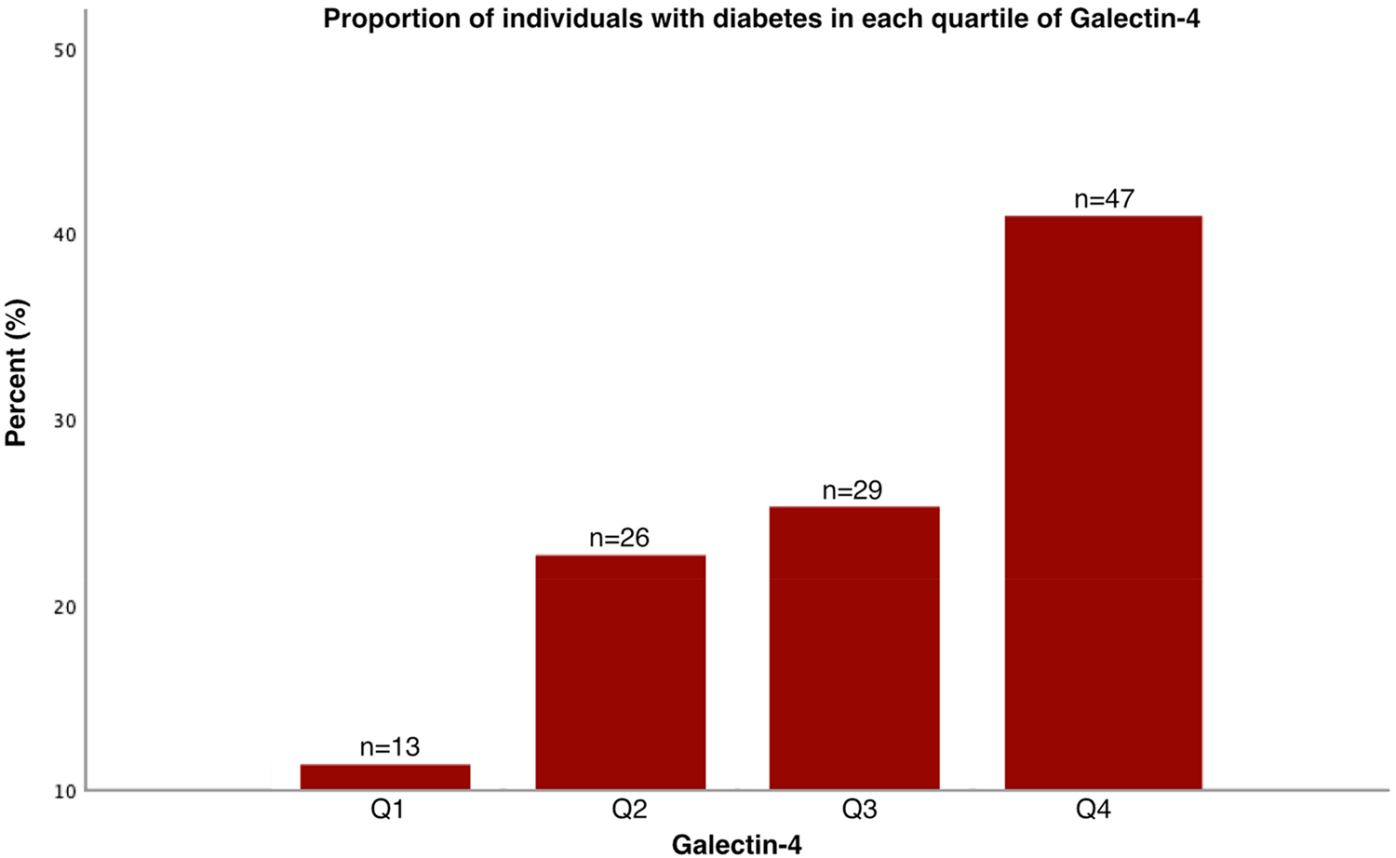
Proportion of individuals with diabetes in each quartile of Galectin-4. *Q1* quartile with lowest Gal-4 levels, *Q4* quartile with highest Gal-4 levels.

In fully adjusted logistic regression models, increased levels of Gal-4 were positively associated with prevalent diabetes (OR 2.60; 1.56–4.32) (Table 3). The proportion of individuals with diabetes increased for every quartile (Q) of Gal-4 (Q1 = lowest Gal-4 levels 11.3%; Q2 22.6%; Q3 25.2%, and Q4 = highest Gal-4 levels 40.9%) (Fig. 1) and, compared to all other quartiles, the odds of having diabetes were the highest in the upper quartile (OR 2.65; 1.33–5.30) (Table 4).

**Table 3:** Galectin-4’s association with diabetes in logistic regression.

Diabetes, n = 309			
115 with diabetes/194 without diabetes			
	OR	CI 95%	*p*
Unadjusted			
Gal-4	2.90	1.93–4.34	** < 0.001**
Model 1			
Gal-4	3.05	2.01–4.63	** < 0.001**
Age	0.99	0.96–1.01	0.174
Sex	0.73	0.42–1.27	0.26
Model 2			
Gal-4	2.60	1.56–4.32	** < 0.001**
Age	0.99	0.96–1.02	0.601
Sex	0.50	0.25–1.01	0.053
BMI	1.12	1.05–1.19	** < 0.001**
Systolic blood pressure	1.00	0.99–1.01	0.482
Triglycerides	0.82	0.36–1.90	0.639
FPG	80.55	19.77–328.25	** < 0.001**
Cystatin C	1.24	0.44–3.55	0.683
Physical activity	1.13	0.61–2.07	0.701

Significance values are in bold.

Values are odds ratios (OR) and 95% confidence intervals (CI95%).

*Gal-4* Galectin-4, *FPG* Fasting plasma glucose.

**Table 4:** Galectin-4’s upper quartile, association with diabetes, compared to all other quartiles in logistic regression.

n = 309			
115 with diabetes/194 without diabetes			
	OR	CI 95%	*p*
Unadjusted			
Gal-4 upper quartile	3.78	2.21–6.47	** < 0.001**
Model 1			
Gal-4 upper quartile	3.86	2.24–6.66	** < 0.001**
Age	0.99	0.97–1.01	0.471
Sex	0.71	0.41–1.23	0.221
Model 2			
Gal-4 upper quartile	2.70	1.35–5.40	**0.005**
Age	1.00	0.97–1.03	0.842
Sex	0.49	0.24–0.99	**0.047**
BMI	1.12	1.05–1.19	** < 0.001**
Systolic blood pressure	1.00	0.99–1.01	0.465
Triglycerides	0.84	0.38–1.87	0.664
FPG	77.7	19.38–311.52	** < 0.001**
Cystatin C	1.67	0.60–4.67	0.326
Physical activity	1.07	0.59–1.95	0.827

Significance values are in bold.

Values are odds ratios (OR) and 95% confidence intervals (CI95%).

*Gal-4* Galectin-4, *FPG* Fasting plasma glucose.

**Figure 2:**
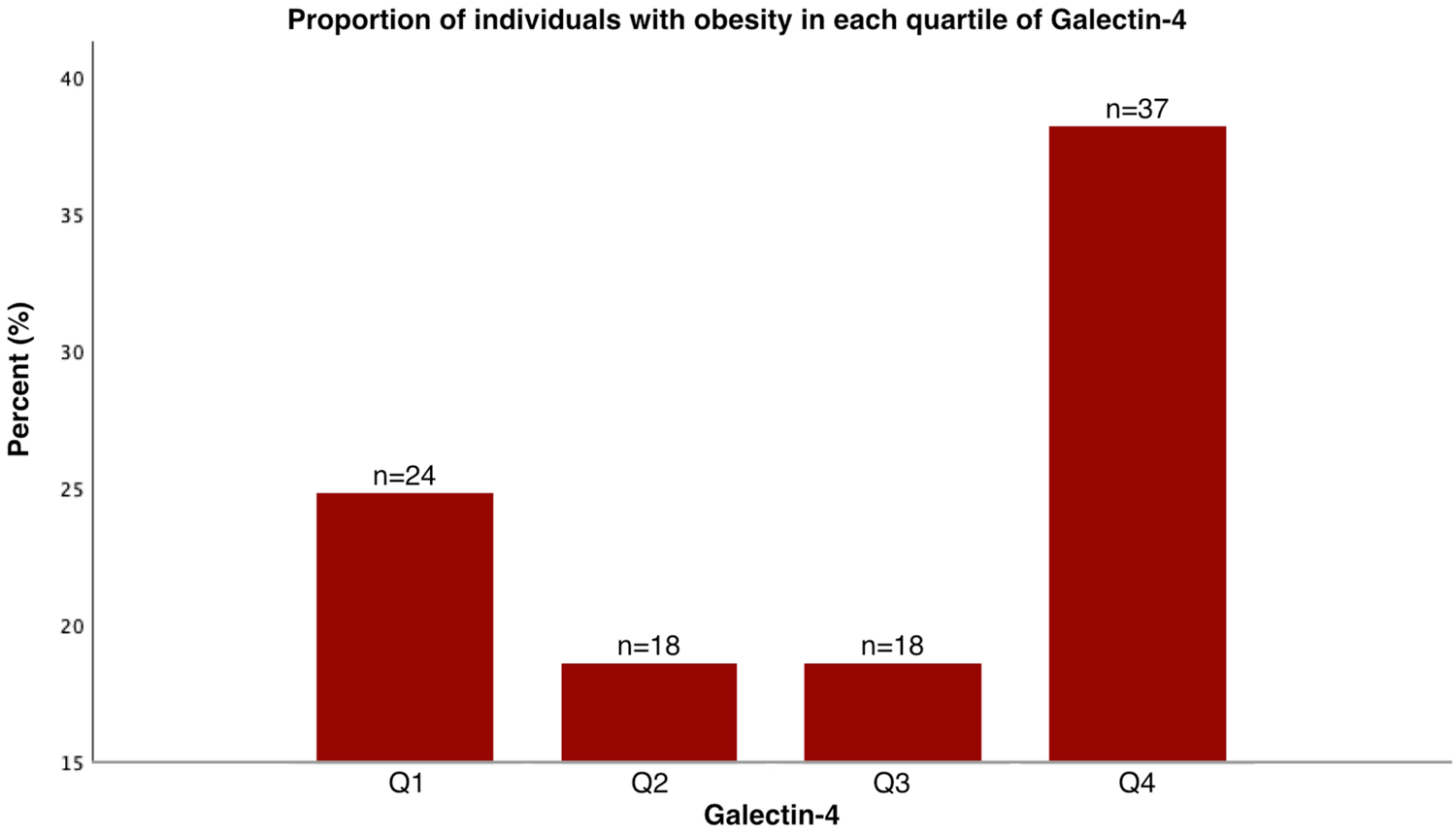
Proportion of individuals with obesity in each quartile of Galectin-4. *Q1* quartile with lowest Gal-4 levels, *Q4* quartile with highest Gal-4 levels.

The proportion of individuals with obesity was the highest in the upper quartile (38.1%) (Fig. 2) and, compared to all other quartiles the odds of having obesity were the highest in the upper quartile (OR 3.38; 1.88–7.20) (Table 5). In fully adjusted logistic regression models, increased levels of Gal-4 were significantly associated with obesity (OR 2.14; 1.34–3.43). An interaction analysis was performed to explore if Gal-4’s association with obesity was moderated by diabetes showing significant interaction between Gal-4 and diabetes; therefore, analyses in subjects with and without diabetes were carried out separately. Upon stratified analysis, an association was observed exclusively among patients with diabetes (OR 2.48; 95% CI 1.09–5.62) as indicated in Table 6. This association was not significant in crude analysis, or in Model 1, but was only shown in Model 2b.

**Table 5:** Galectin-4’s upper quartile, association with obesity, compared to all other quartiles in logistic regression.

n = 309			
97 with obesity/212 without obesity			
	OR	CI 95%	*p*
Unadjusted			
Gal-4 upper quartile	2.65	1.55–4.53	** < 0.001**
Model 1			
Gal-4 upper quartile	3.48	1.95–6.20	** < 0.001**
Age	0.94	0.92–0.96	** < 0.001**
Sex	1.89	1.06–3.38	**0.032**
Model 2			
Gal-4 upper quartile	3.68	1.88–7.20	** < 0.001**
Age	0.93	0.91–0.96	** < 0.001**
Sex	1.78	0.96–3.30	0.069
Systolic blood pressure	1.01	1.00–1.02	**0.008**
Triglycerides	1.53	0.74–3.17	0.242
FPG	6.05	2.16–16.93	** < 0.001**
Cystatin C	0.77	0.28–2.09	0.604
Physical activity	0.87	0.50–1.58	0.654

Significance values are in bold.

Values are odds ratios (OR) and 95% confidence intervals (CI95%).

*Gal-4* Galectin-4, *FPG* Fasting plasma glucose.

**Table 6:** Galectin-4’s association with obesity (BMI ≥ 30) in logistic regression.

	Total n = 309			Individuals with diabetes n = 115			Individuals without diabetes n = 194		
	OR	CI (95%)	*p*	OR	CI (95%)	*p*	OR	CI (95%)	*p*
Unadjusted									
Gal-4	1.56	1.07–2.27	0.022	1.49	0.82–2.73	0.194	0.83	0.46–1.52	0.552
Model 1									
Gal-4	2.07	1.37–3.13	** < 0.001**	1.78	0.89–3.55	0.101	1.16	0.62–2.21	0.64
Age	0.94	0.91–0.96	** < 0.001**	0.90	0.85–0.95	** < 0.001**	0.95	0.92–0.98	**0.001**
Sex	1.89	1.06–3.36	**0.031**	4.06	1.47–11.20	**0.007**	1.60	0.71–3.16	0.258
Model 2									
Gal-4	2.14	1.34–3.43	**0.001**	2.48	1.09–5.62	**0.03**	1.33	0.68–2.62	0.402
Age	0.93	0.90–0.98	** < 0.001**	0.87	0.83–0.94	** < 0.001**	0.95	0.91–0.98	0**.003**
Sex	1.82	0.98–3.38	0.057	3.73	1.27–10.94	**0.017**	1.57	0.66–3.73	0.303
Systolic blood pressure	1.02	1.00–1.02	**0.009**	1.02	1.00–1.04	0.080	1.01	1.00–1.03	**0.031**
Triglycerides	1.55	0.75–3.17	0.234	1.04	0.36–2.99	0.938	1.97	0.70–5.61	0.202
FPG	6.43	2.29–18.04	** < 0.001**	2.26	0.55–9.26	0.258	3.23	0.37–28.25	0.289
Cystatin C	0.80	0.30–2.17	0.663	0.34	0.06–1.90	0.221	0.98	0.24–3.99	0.972
Physical activity	0.95	0.54–1.67	0.86	1.03	0.42–2.54	0.945	0.97	0.43–2.19	0.946

Significance values are in bold.

Values are odds ratios (OR) and 95% confidence intervals (CI95%).

*Gal-4* Galectin-4, *FPG* Fasting plasma glucose.

Gal-4 exhibited no discernible associations with heart failure phenotype (HFpEF vs HFrEF) or LVH (Supplementary Tables S1, S2). Gal-4 was significantly associated with IHD (n = 126, 40.8%) and severity of HF symptoms (NYHA-class III-IV, n = 263, 85.1%) in unadjusted and Model 1 adjusted analyses, but the association was attenuated upon Model 2a adjustment (Supplementary Tables S3, S4).

### Stratified analyses

When examining Gal-4's relationship with diabetes in models stratified by IHD/non-IHD and HFpEF/HFrEF, the analysis revealed significant associations. Specifically, Gal-4 showed a significant association with diabetes in individuals with HFrEF (n = 160), but not in those with HFpEF (n = 70, Supplementary Table S5). Additionally, Gal-4 demonstrated a significant association with diabetes in both patients with and without IHD (Supplementary Table S6).

## Discussion

Among HF patients, elevated Gal-4 levels were notably linked with existing diabetes and higher glucose levels, along with the incretin GIP. Moreover, increased Gal-4 levels were significantly associated with increased likelihood of obesity, but this association was observed exclusively in diabetic patients. Furthermore, Gal-4 levels were elevated in patients with ischemic heart disease (IHD). In stratified analyses, it was revealed that Gal-4 exhibited distinct associations with both diabetes and obesity in patients specifically diagnosed with HFrEF. These results confirm Gal-4’s role in concomitant diabetes and HF while revealing new findings of Gal-4’s role in obesity, and link with GIP, in HF patients.

Gal-4, a member of the β-galactoside-binding protein family, contains two carbohydrate-recognition domains within a single peptide chain. Predominantly expressed in the epithelial cells of the intestinal tract, it is secreted into the extracellular space, where it is localized to the brush border. Although the two domains share 40% similarity in amino acid sequence, they exhibit distinct binding properties to various ligands. This unique feature allows Gal-4 to serve as a crucial crosslinker and regulator in numerous biological processes^6^. Research highlights its significant roles in stabilizing lipid rafts^8^, facilitating protein apical trafficking^22^, participating in wound healing^23^, influencing intestinal inflammation^24^, and contributing to tumour progression^25^, among other functions. The last decade, Gal-4 has also been implicated in HF^10^ and diabetes^11–15^.

The observed association between Gal-4 and IHD in this study, although attenuated upon full adjustment, along with the observation of higher Gal-4 levels in subjects with IHD likely arises from a complex interplay of biological processes. Gal-4 can bind to CD14 on monocytes and induce them to differentiate into macrophages through the MAPK signalling pathway, thus regulating inflammation^6^, and may play a role in the chronic low-grade inflammation observed in IHD^26,27^, contributing to atherosclerosis and plaque formation. Indeed, we previously showed that Gal-4 levels are increased in mice and humans with prevalent stroke^28^. There is limited research on whether Gal-4 specifically is involved in fibrosis. However, as indicated in a review by Yu et al., it is believed that galectins have similar and significant roles in the fibrotic process by promoting myofibroblasts to secrete extracellular matrix^23^. This is particularly pertinent, as fibrotic processes are often engaged to replace damaged myocardial tissue in IHD. Fibrotic scars in cardiac muscle primarily develop following a myocardial infarction^29^. Nevertheless, other conditions, including hypertensive heart disease and diabetic hypertrophic cardiomyopathy can also contribute to the occurrence of cardiac fibrosis^30^.

On the other hand, diabetes exerts a direct impact on the myocardium,^20^ and is known to impair microvascular function^31^, potentially rendering the myocardium more susceptible to ischemic damage^32^. Gal-4’s association with glucose metabolism, diabetes and obesity as seen in our study might be significant in the context of IHD, which is often accompanied by metabolic perturbations^33–35^.

The association between Gal-4 and diabetes in subjects with HFrEF appears to be influenced by several intricate factors. Previous studies have shown that inflammatory markers were more strongly correlated with HFpEF, while biomechanical cardiac stress markers like BNP showed a stronger association with HFrEF^36^. Given our discovery of associations between Gal-4 and NT-proBNP, ST2, and Gal-3, all of which serve as biomarkers indicating the severity of HF^37^,we suggest a potential link between Gal-4 expression and the severity of HF. This finding may imply that Gal-4 plays a role in the pathophysiological processes underlying HF progression.

Additionally, Gal-4's involvement in diabetes^11–15^ aligns with the metabolic abnormalities often seen in HF, potentially reinforcing its association with diabetes. The interplay of various factors may also contribute to this observed association. However, the observation that Gal-4 is associated with diabetes exclusively in subjects with HFrEF could potentially be influenced by limitations in statistical power in stratified analyses, given the relatively small cohort size of 70 individuals with HFpEF, of which 27 (38.6%) had diabetes. Nevertheless, further mechanistic studies are imperative to corroborate and comprehensively understand the nuanced association between Gal-4 and diabetes within distinct HF phenotypes.

Under normal conditions, Gal-4 is a protein found intracellularly, within the gastrointestinal tract, or on the brush border of enterocytes. However, here we found increased levels of Gal-4 in plasma. We speculate whether this is due to an impaired intestinal barrier function. Increased levels of Gal-4 have been found in the lamina propria of mice with a damaged colon^38^, as well as in the serum of neonates with gastrointestinal emergencies^39^. Both studies implied that the elevated levels of Gal-4 were due to a damaged digestive tract. It has been suggested that HF may lead to gastrointestinal hypoperfusion, causing wall oedema and impaired intestinal barrier function.

Gal-4 facilitates protein transport, including DPP4, in enterocytes. DPP4 inhibitors in diabetes treatment may pose a HF risk^40^. Inflammation may alter Gal-4's intracellular role, impacting DPP4 function and elevating GIP and GLP-1 levels. Interestingly, in this study, we found Gal-4 to be significantly associated with GIP, as well as with diabetes. Whether the increased levels of circulating Gal-4 found in this study are related to a decreased ability to transport DPP4 and thereby increasing the odds of diabetes is beyond the scope of this study.

As for Mendelian randomisation studies, adequately powered studies investigating Gal-4 and diabetes studies are unfortunately lacking.

### Limitations

The results of the study should be interpreted in the light of the study limitations. One limitation of our study is that we lacked data on echo parameters for 26% of the study population, potentially reducing statistical power. Data is lacking to this extent because patients admitted for HF on general medicine wards were less likely to undergo echocardiography upon hospital admission compared to those admitted to specialized cardiology departments. Nevertheless, it's likely that this could lead to an underestimation of the observed effects, rather than an overestimation. The application of the results on all HF populations is limited as data was collected at a single regional hospital, and participants were of mainly white decent. The cross-sectional design of the study limits any causal inference.

## Conclusions

Among heart failure patients, elevated Gal-4 levels were associated with higher GIP levels. Moreover, increased Gal-4 levels were associated with an increased likelihood of diabetes, and obesity and this association was particularly pronounced in individuals with heart failure characterized by reduced ejection fraction. Furthermore, Gal-4 levels were significantly higher in heart failure patients with diabetes, obesity, and those with ischemic heart failure aetiology, compared to those without. These findings highlight the potential relevance of Gal-4 in the context of heart failure and its related metabolic and cardiovascular aspects. Further research is warranted to elucidate the mechanistic underpinnings and clinical implications of these associations.

### Supplementary Information

Supplementary Tables.

## Data Availability

The data that support the findings of this study are available upon a reasonable request to MM but restrictions apply to the availability of these data, which were used under license for the current study, and so are not publicly available due to ethical and legal restrictions related to the Swedish Biobanks in Medical Care Act (2002:297) and the Personal Data Act (1998:204).

## Abbreviations

DPP4: Dipeptidyl peptidase-4
Gal-4: Galectin-4
GIP: Glucose-dependent insulinotropic polypeptide
HF: Heart failure
