# Supplementary material for: Galectin-4 is associated with diabetes and obesity in a heart failure population
Source: Sci Rep. 2023 Nov 20;13:20285. doi: 10.1038/s41598-023-47426-9 (PMC10662206; doi:10.1038/s41598-023-47426-9)
Supplement: Supplementary file 1 — Supplementary Tables. [file 41598_2023_47426_MOESM1_ESM.docx]

**SUPPLEMENTARY MATERIAL**

**Galectin-4 is associated with diabetes and obesity in a heart failure population**

All values are odds ratios with 95% confidence intervals.

**Supplementary Table S1.** Galectin 4’s association with heart failure phenotype (HFpEF vs HFrEF)

|  |  | |
| --- | --- | --- |
| **UNADJUSTED** | OR (95%CI) | p |
| Gal-4 | 1.30 (0.84-2.01) | 0.238 |

**Supplementary Table S2**. Galectin 4’s association with left ventricular hypertrophy

| UNADJUSTED | OR (95%CI) | p |
| --- | --- | --- |
| Gal-4 | 1.01 (0.66-1.55) | 0.954 |

**Supplementary Table S3**. Galectin-4’s association with heart failure aetiology (ischemic heart disease vs non-ischemic heart disease)

| UNADJUSTED | OR (95%CI) | p |
| --- | --- | --- |
| Gal-4 | 1.51 (1.05-2.18) | 0.026 |
|  |  |  |
| MODEL 1 |  |  |
| Gal-4 | 1.47 (1.01-2.13) | 0.044 |
| Age | 1.01 (0.99-1.03) | 0.424 |
| Sex | 0.66 (0.39-1.11) | 0.116 |
|  |  |  |
| MODEL 2 |  |  |
| Gal-4 | 1.25 (0.82-1.91) | 0.297 |
| Age | 1.02 (0.99-1.05) | 0.151 |
| Sex | 0.64 (0.37-1.12) | 0.121 |
| BMI | 1.02 (0.98-1.07) | 0.333 |
| SBP | 0.99 (0.98-1.00) | 0.089 |
| Triglycerides | 2.14 (1.10-4.19) | 0.025 |
| FPG | 3.26 (1.27-8.36) | 0.014 |
| Cystatin C | 1.01 (0.43-2.39) | 0.983 |
| Physical activity | 1.30 (0.79-2.15) | 0.299 |

**Supplementary Table S4**. Galectin 4’s association with severity of heart failure symptoms (NYHA-class III-IV)

| UNADJUSTED | OR (95%CI) | p |
| --- | --- | --- |
| Gal-4 | 2.06 (1.20-3.55) | 0.009 |
|  |  |  |
| MODEL 1 |  |  |
| Gal-4 | 1.87 (1.08-3.25) | 0.026 |
| Age | 1.02 (0.99-1.05) | 0.229 |
| Sex | 2.21 (0.93-5.27) | 0.073 |
|  |  |  |
| MODEL 2 |  |  |
| Gal-4 | 1.09 (0.58-2.04) | 0.785 |
| Age | 1.01 (0.98-1.05) | 0.451 |
| Sex | 2.56 (1.02-6.43) | 0.046 |
| BMI | 1.12 (1.04-1.22) | 0.005 |
| SBP | 0.99 (0.98-1.00) | 0.052 |
| Triglycerides | 0.74 (0.29-1.85) | 0.516 |
| FPG | 1.91 (0.42-8.59) | 0.400 |
| Cystatin C | 5.64 (1.35-23.56) | 0.018 |
| Physical activity | 0.57 (0.28-1.15) | 0.116 |

**Supplementary Table S5**. Galectin 4’s association with diabetes in analyses stratified on HFrEF vs HFpEF

|  | **HFrEF** | | **HFpEF** |  |
| --- | --- | --- | --- | --- |
| **UNADJUSTED** | OR (95%CI) | p | OR (95%CI) | p |
| Gal-4 | 3.26 (1.88-5.66) | <0.001 | 1.96 (0.75-5.10) | 0.169 |
|  |  |  |  |  |
| **MODEL 1** |  |  |  |  |
| Gal-4 | 3.15 (1.78-5.55) | <0.001 |  |  |
| Age | 1.01 (0.98-1.04) | 0.510 |  |  |
| Sex | 0.48 (0.19-1.23) | 0.127 |  |  |
|  |  |  |  |  |
| **MODEL 2** | (Lower-Upper) |  |  |  |
| Gal-4 | 2.97 (1.46-6.03) | 0.003 |  |  |
| Age | 1.01 (0.97-1.06) | 0.668 |  |  |
| Sex | 0.45 (0.14-1.40) | 0.166 |  |  |
| BMI | 1.06 (0.97-1.17) | 0.178 |  |  |
| SBP | 0.99 (0.98-1.01) | 0.443 |  |  |
| Triglycerides | 0.51 (0.17-1.59) | 0.247 |  |  |
| FPG | 2.79 (1.35-5.74) | 0.005 |  |  |
| Cystatin C | 1.31 (0.32-5.39) | 0.708 |  |  |
| Physical activity | 1.22 (0.54-2.77) | 0.630 |  |  |

**Supplementary Table S6**. Galectin-4’s association with diabetes in analyses stratified on ischemic heart disease vs non-ischemic heart disease

|  | Non-IHD | | IHD | |
| --- | --- | --- | --- | --- |
| **UNADJUSTED** | OR (95%CI) | p | OR (95%CI) | p |
| Gal-4 | 2.73 (1.60-4.67) | <0.001 | 3.21 (1.62-6.35) | 0.001 |
|  |  |  |  |  |
| **MODEL 1** |  |  |  |  |
| Gal-4 | 2.97 (1.70-5.21) | <0.001 | 3.52 (1.74-7.11) | <0.001 |
| Age | 0.99 (0.96-1.02) | 0.473 | 0.97 (0.93-1.01) | 0.105 |
| Sex | 0.52 (0.24-1.14) | 0.105 | 1.66 (0.68-4.09) | 0.268 |
|  |  |  |  |  |
| **MODEL 2** |  |  |  |  |
| Gal-4 | 3.62 (1.69-7.75) | 0.001 | 3.18 (1.33-7.60) | 0.009 |
| Age | 0.99 (0.94-1.04) | 0.688 | 0.97 (0.92-1.02) | 0.242 |
| Sex | 0.30 (0.10-0.88) | 0.028 | 1.10 (0.35-3.40) | 0.871 |
| BMI | 1.19 (1.08-1.32) | 0.000 | 1.06 (0.97-1.15) | 0.180 |
| SBP | 1.00 (0.98-1.01) | 0.769 | 0.99 (0.98-1.01) | 0.577 |
| Triglycerides | 0.32 (0.08-1.29) | 0.109 | 1.43 (0.45-4.53) | 0.538 |
| FPG | 3.71 (2.10-6.55) | 0.000 | 3.11 (1.66-5.81) | 0.000 |
| Cystatin C | 1.21 (0.29-5.07) | 0.796 | 0.89 (0.15-5.10) | 0.892 |
| Physical activity | 0.53 (0.20-1.38) | 0.194 | 2.79 (1.06-7.36) | 0.038 |
